# DAZL Relieves miRNA-Mediated Repression of Germline mRNAs by Controlling Poly(A) Tail Length in Zebrafish

**DOI:** 10.1371/journal.pone.0007513

**Published:** 2009-10-19

**Authors:** Yasuaki Takeda, Yuichiro Mishima, Toshinobu Fujiwara, Hiroshi Sakamoto, Kunio Inoue

**Affiliations:** 1 Department of Biology, Graduate School of Science, Kobe University, Kobe, Japan; 2 Department of Chemical Science and Engineering, Graduate School of Engineering, Kobe University, Kobe, Japan; Harvard University, United States of America

## Abstract

**Background:**

During zebrafish embryogenesis, microRNA (miRNA) miR-430 contributes to restrict Nanos1 and TDRD7 to primordial germ cells (PGCs) by inducing mRNA deadenylation, mRNA degradation, and translational repression of *nanos1* and *tdrd7* mRNAs in somatic cells. The *nanos1* and *tdrd7* 3′UTRs include *cis*-acting elements that allow activity in PGCs even in the presence of miRNA-mediated repression.

**Methodology/Principal Findings:**

Using a GFP reporter mRNA that was fused with *tdrd7* 3′UTR, we show that a germline-specific RNA-binding protein DAZ-like (DAZL) can relieve the miR-430-mediated repression of *tdrd7* mRNA by inducing poly(A) tail elongation (polyadenylation) in zebrafish. We also show that DAZL enhances protein synthesis via the 3′UTR of *dazl* mRNA, another germline mRNA targeted by miR-430.

**Conclusions/Significance:**

Our present study indicated that DAZL acts as an “anti-miRNA factor” during vertebrate germ cell development. Our data also suggested that miRNA-mediated regulation can be modulated on specific target mRNAs through the poly(A) tail control.

## Introduction

Post-transcrptional regulation plays a crucial role in germ cell development. In zebrafish, primordial germ cells (PGCs) are determined by the incorporation of a specific maternal cytoplasm called germ plasm, which forms at the ends of cleavage planes in four-cell stage embryos [1], [2]. Several maternal mRNAs, such as *vasa* [1], *nanos1* [3], *dead-end (dnd)* [4], and *Tudor domain-containing protein 7 (tdrd7)* [5], are known to be localized to the germ plasm (hereinafter called germ plasm mRNAs). However, a fraction of germ plasm mRNAs is detected throughout the blastomeres during cleavage stages. Such unlocalized mRNAs are subsequently incorporated into somatic cells as well as PGCs. It has been shown that *vasa*, *nanos1*, and *tdrd7* mRNAs are rapidly degraded in somatic cells but are stabilized in PGCs in a process mediated by *cis*-acting elements in their 3′ untranslated regions (3′ UTRs) [3], [5], [6], [7]. Translation control also contributes to the restriction of NANOS expression to PGCs [3]. Our previous study showed that microRNA (miRNA) miR-430, which is abundantly expressed from the onset of zygotic gene expression [8], targets the 3′UTRs of *nanos1* and *tdrd7* to induce mRNA deadenylation, mRNA degradation, and translational repression in somatic cells [5]. Importantly, miR-430 is also expressed in PGCs, and other target mRNAs of miR-430 are equally susceptible to repression in somatic cells and PGCs [5], [9]. The *nanos1* and *tdrd7* 3′UTRs include *cis*-acting elements that allow activity in PGCs even in the presence of miRNA-mediated repression [5]. Thus miR-430-mediated repression and the activation of germ plasm mRNAs play important roles in germline/somatic cell distinctions in zebrafish embryos.

Recently, Kedde et al. reported that a germline-specific RNA-binding protein DND counteracts the function of several miRNAs by binding mRNAs and prohibiting them from associating with their target sites [10]. In zebrafish PGCs, DND alleviates miR-430 repression of *nanos1* and *tdrd7*. In the present study, we assumed that another RNA-binding protein, DAZL, can also relieve the miR-430-mediated repression of germ plasm mRNAs in zebrafish, for two reasons. First, as an evolutionarily conserved RNA-binding protein, DAZL plays a key role in germ cell development in animals such as *Drosophila*, *C. elegans*, *Xenopus*, and mouse [11]. In *Xenopus* and zebrafish, *dazl* mRNA is present in the germ plasm and in PGCs during early embryogenesis [2], [12]. Second, DAZL activates the translation efficiency of the target mRNAs through direct binding to *cis*-elements in their 3′UTRs [13]. Moreover, *Xenopus* DAZL interacts with poly(A)-binding proteins (PABPs), which are critical for the initiation of translation [14].

Using a GFP reporter mRNA that was fused with *tdrd7* 3′UTR, we show here that DAZL antagonizes miR-430-mediated repression of the *tdrd7* mRNA in zebrafish embryos. Moreover, the addition of DAZL-binding elements to the synthetic miR-430 target mRNA led to mRNA stabilization in a PGC-specific manner in embryos. We also show that DAZL can enhance protein synthesis via the 3′UTR of *dazl* mRNA which is localized to PGCs and targeted by miR-430. To our surprise, we found that DAZL induces polyadenylation of the reporter mRNA irrespective of the function of miRNA. Taken together, these results indicated that DAZL acts as an “anti-miRNA factor” during vertebrate germ cell development. Our data also suggested that miRNA-mediated regulation can be modulated on specific target mRNAs through the poly(A) tail control.

## Results

### DAZL activates *tdrd7* expression through mRNA stabilization

**Figure 1:**
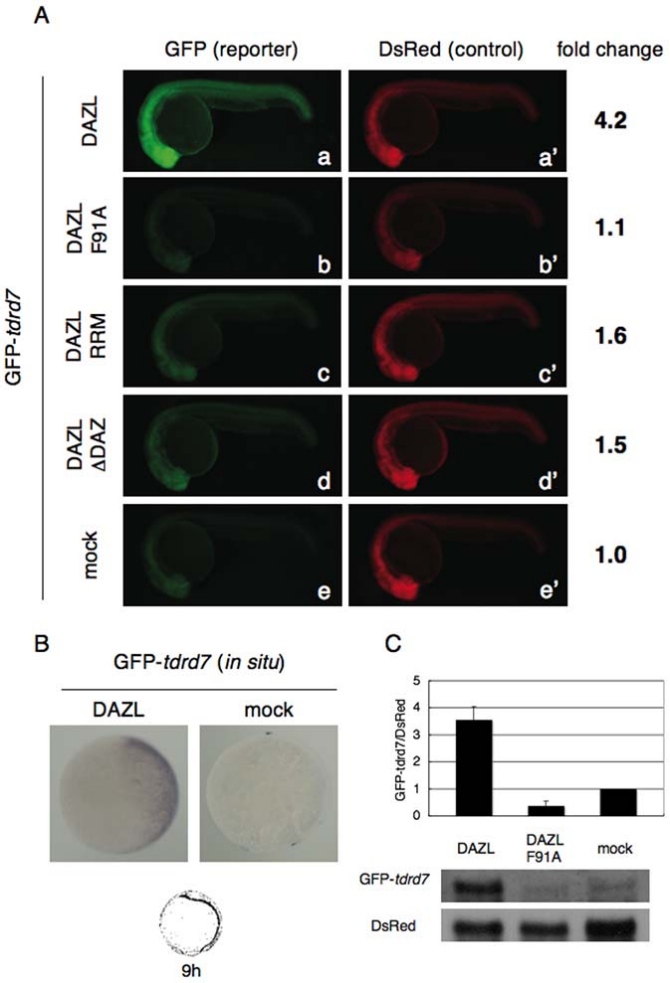
Activation of GFP-*tdrd7* expression by DAZL. (A) GFP-*tdrd7* and DsRed mRNAs were co-injected with the mRNAs encoding Myc-tagged DAZL, DAZL F91A, DAZL RRM, or DAZL ΔDAZ mRNA, or without any *dazl* mRNA (mock) at the one-cell stage. GFP and DsRed expression were analyzed at 24 hr post-fertilization (hpf). Fold change of normalized GFP fluorescence relative to mock control is shown on the right. (B) *In situ* hybridization of the injected embryos at 80% epiboly with an antisense probe for GFP. GFP-*tdrd7* and DsRed mRNAs were injected with or without the mRNA encoding Myc-DAZL. (C) Northern blotting of the injected GFP-*tdrd7* and DsRed mRNAs. The ratio of GFP mRNA to DsRed mRNA from three independent experiments is shown.

To clarify whether or not DAZL enhances *tdrd7* expression, we performed injection experiments of GFP reporter mRNA containing *tdrd7* 3′UTR (GFP-*tdrd7*) [5] (Fig. 1). As an internal control, DsRed reporter mRNA was co-injected (Fig. 1A). When GFP-*tdrd7* and DsRed mRNAs were injected, GFP expression was barely detectable in somatic cells at 24 hr post-fertilization (hpf), due to the repression by miR-430 [5] (Fig. 1A, panel e). In contrast, when wild-type DAZL was co-expressed (see Supplementary Fig. S1), we observed strong enhancement of GFP expression (4.2-fold compared to mock control) throughout the embryos, although DsRed expression was not affected (Fig. 1A, panel a). The mutant DAZL protein, DAZL F91A, which lacks RNA-binding activity because of the amino acid substitution in the RNA-recognition motif (RRM) [15], did not promote GFP-*tdrd7* mRNA expression (Fig. 1A, panel b). In addition, we tested two types of truncated DAZL protein that cannot activate translation of target mRNAs: DAZL RRM possesses only the N-terminal portion containing RRM, whereas DAZL ΔDAZ lacks a conserved DAZ motif [15] (Fig. S1). We found that these truncated DAZL proteins enhanced GFP-*tdrd7* expression only weakly (∼1.6-fold, Fig. 1A, panels c and d). These results showed that DAZL can activate protein synthesis through the 3′UTR of *tdrd7* mRNA targeted by miR-430, and that the RRM and DAZ motifs are involved in the activation.

Efficiency of protein synthesis is often controlled by mRNA stability (stabilization/degradation) and/or translation efficiency (activation/repression) [16], [17]. In zebrafish embryos, miR-430 enhances the degradation of target mRNAs [9]. Therefore, we examined whether or not DAZL protein affects the stability of GFP-*tdrd7* mRNA. *In situ* hybridization of the embryos injected with GFP-*tdrd7* mRNA was performed at 80%-epiboly (∼8 hpf), since miR-430-mediated mRNA degradation can be observed immediately after the onset of zygotic expression (around 4–8 hpf) [5]. As a result, we found that the reporter mRNA abounded in the presence of DAZL protein, as compared with mock control (Fig. 1B). Northern analysis revealed that the amount of injected GFP-*tdrd7* mRNA was increased more than 3-fold by DAZL expression (Fig. 1C). These results indicated that DAZL activates *tdrd7* expression at least in part by enhancing mRNA stability.

### The *tdrd7* 3′UTR contains *cis*-elements for the activation by DAZL protein

We examined whether DAZL's effect is specific to the miR-430 target site in the context of *tdrd7* 3′UTR. The miR-204 target site (IPT^miR204^) was inserted into the 3′UTR of the GFP-*tdrd7* construct, in which the miR-430 target sites [5] had been disrupted by base substitutions (Fig. S2, panels A and B). miR-204 is not strongly expressed during early embryogenesis and therefore we could mimic the ubiquitous expression of miR-430 by injecting miR-204 duplex [5]. We found that somatic GFP expression from the reporter mRNA was inhibited by miR-204 co-injected into the embryo. In contrast, DAZL overexpression caused strong GFP expression from the reporter mRNA even in the presence of miR-204 (3.3-fold, Fig. S2, panel C). These results suggested that the primary sequence of the miRNA target site or the local secondary structure containing the target site is not important for the relief of miRNA function, and that the *tdrd7* 3′UTR contains some *cis*-acting element(s) responsible for the regulation by DAZL.

**Figure 2:**
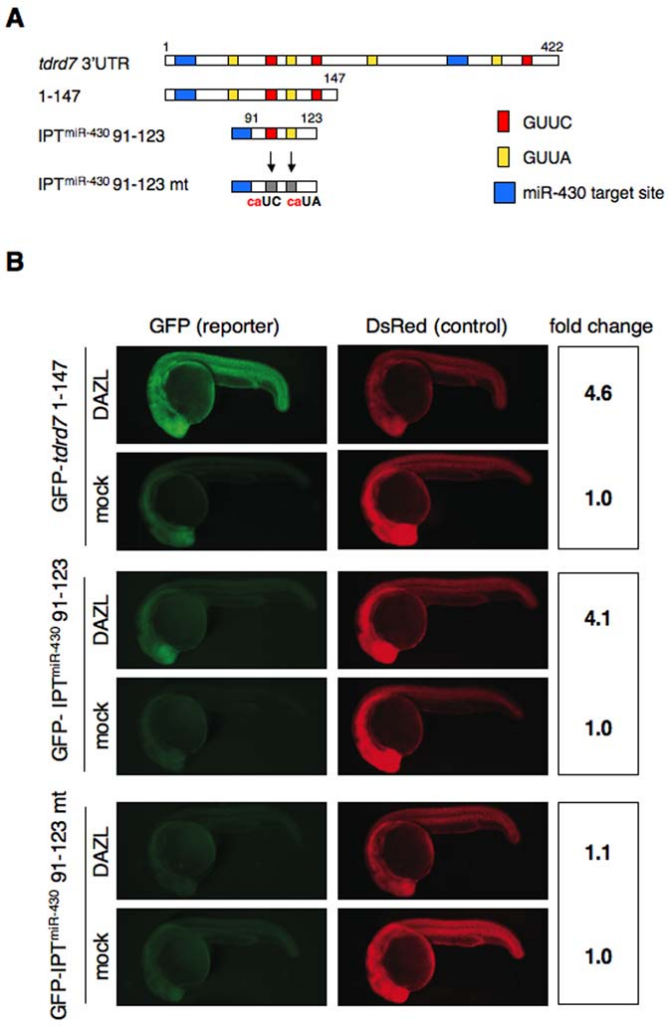
Identification of the *cis*-element required for the regulation by DAZL. (A) Schematic representation of *tdrd7* 3′UTR mutant constructs. Putative DAZL-binding sites (GUUC; red, GUUA; yellow), miR-430 target sites (blue), and mutated DAZL-binding sites (gray) are indicated. Nucleotide positions relative to the stop codon are shown above. The exogenous miR-430 target site (IPT^miR-430^) in the IPT^miR-430^ 91–123 and IPT^miR-430^ 91–123 mt constructs were derived from the *nanos1* 3′UTR [5]. (B) Injection experiments of mutant GFP-*tdrd7* mRNA and DsRed mRNA with or without the mRNA encoding Myc-DAZL. GFP and DsRed were analyzed at 24 hpf. Fold change of normalized GFP fluorescence relative to mock control is shown on the right.

Previous studies showed that the DAZ family proteins bind to the *cis* elements in 3′UTR to activate the translation of target mRNAs: zebrafish DAZL binds to the GUUC element [15], while human and mouse DAZL bind to U-rich elements such as U2-10(G/C)U2-10 and (G/C)Un [18], [19], [20], [21]. We examined whether *tdrd7* 3′UTR has such a *cis*-regulatory element responsible for the enhanced expression induced by DAZL. When a GFP reporter mRNA having the 5′ half of *tdrd7* 3′UTR (corresponding to the nucleotides +1–147 relative to the stop codon) was tested, DAZL strongly activated GFP expression (4.6-fold, Fig. 2B), suggesting that this region includes a *cis*-element(s) for DAZL. Furthermore, DAZL activated the expression of GFP-IPT^miR-430^ 91–123 mRNA that contains a portion of the *tdrd7* 3′UTR (corresponding to the nucleoteides +91–123 relative to the stop codon) fused with a single copy of the exogenous miR-430 target site (IPT^miR-430^) (4.1-fold, Fig. 2B). This portion of the 3′UTR contains the GUUC element and its related sequence, GUUA. When we examined a reporter mRNA, GFP-IPT^miR-430^ 91–123 mt, in which we had introduced the base substitutions into the GUUC and GUUA elements, GFP expression was not enhanced even in the presence of DAZL (1.1-fold, Fig. 2B). These results strongly suggested that DAZL activates *tdrd7* expression via binding to the *cis*-elements in the 3′UTR. The *tdrd7* 3′UTR contains additional copies of the GUUC element as well as U-rich sequences outside region +91–123 (Fig. 2A). Although we have not examined the role of these elements, it is possible that DAZL acts on such elements to enhance the activation of *tdrd7* expression.

### DAZL-binding element is sufficient for the PGC-specific activation of miR-430 target mRNA

**Figure 3:**
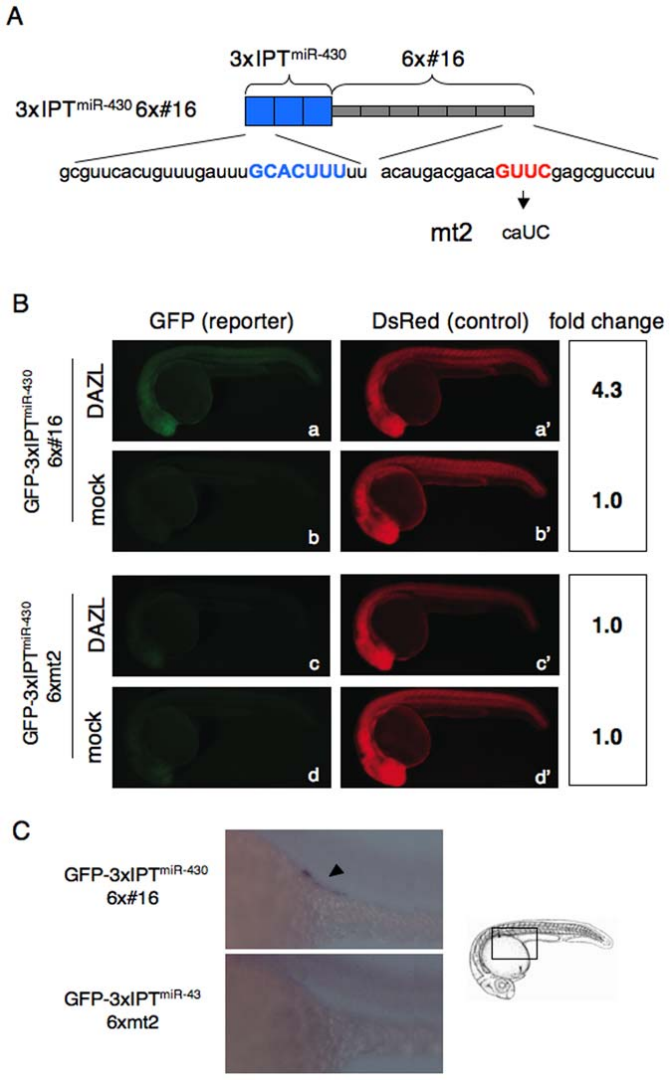
DAZL relieves the miR-430-mediated repression via binding to the *cis*-element GUUC. (A) Schematic representation of the 3′UTR of GFP-3xIPT^miR-430^ 6x#16 and GFP-3xIPT^miR-430^ 6xmt2. Sequences of IPT^miR-430^ and #16 are shown below. The sequence that basepairs with miR-430 seed (blue) and the DAZL-binding sequence (red) are indicated. (B) Injection experiments of GFP-3xIPT^miR-430^ 6x#16 (a and b) or GFP-3xIPT^miR-430^ 6xmt2 (c and d) with or without the mRNA encoding Myc-DAZL. DsRed mRNA was co-injected. GFP and DsRed expression were analyzed at 24 hpf. Fold change of normalized GFP fluorescence relative to mock control is shown on the right. (C) *In situ* hybridization of the embryos injected with GFP-3xIPT^miR-430^ 6x#16 or GFP-3xIPT^miR-430^ 6xmt2 with a GFP probe at 24 hpf. The boxed region in the schematic representation was enlarged. Arrowhead indicates the PGCs.

Expression of the reporter mRNA, GFP-3xIPT^miR-430^, containing three copies of synthetic miR-430 target site (IPT^miR-430^) that is derived from *nanos1* 3′UTR is completely repressed by miR-430 not only in somatic cells but also in PGCs [5], [9]. To further examine whether or not direct binding of DAZL to the *cis*-element is sufficient for the relief of miRNA-mediated repression, we tested a reporter mRNA, GFP-3xIPT^miR-430^ 6x#16, containing three copies of the miR-430 target site fused with six copies of the *in vitro* selected DAZL-binding sequence, #16, that possessed the GUUC element [15] (Fig. 3A). We found that DAZL enhanced GFP expression from the reporter mRNA (4.3-fold, Fig. 3B). In contrast, GFP expression of a reporter mRNA (GFP-3xIPT^miR-430^6xmt2), containing miR-430 target sites with the mutant elements of #16 that do not bind to DAZL [15], was not largely affected by DAZL (1.0-fold, Fig. 3B). These results led us to conclude that DAZL binds to the *cis*-elements in the 3′UTR and cancels the miR-430-mediated repression of *tdrd7*.

To determine whether miR-430-mediated repression is relieved endogenously through the DAZL-binding sequence, we performed *in situ* hybridization of embryos injected with GFP-3xIPT^miR-430^ 6x#16 mRNA. As a result, the mRNA was detected in a PGC-specific manner in the absence of exogenous DAZL, while GFP-3xIPT^miR-430^+6xmt2 mRNA was undetectable throughout the embryo (Fig. 3C). These results suggested that endogenous DAZL protein cancels the repressive effect of miR-430 on *tdrd7* mRNA in PGCs.

### Induction of mRNA polyadenylation by DAZL

**Figure 4:**
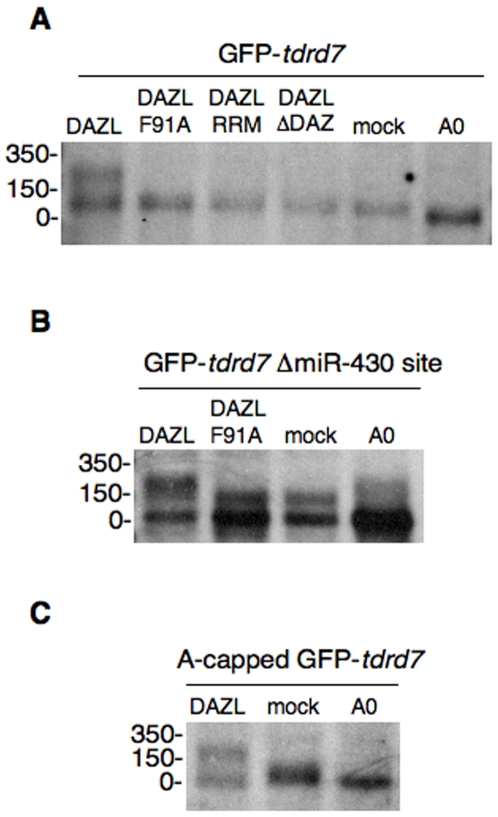
DAZL induces mRNA polyadenylation. Poly(A) length of GFP-*tdrd7* (A), GFP-*tdrd7* ΔmiR-430 site (B), or A-capped GFP-*tdrd7* (C) in the presence or absence of DAZL proteins was analyzed by RNase H-poly(A) assay at 6 hpf. (A0) shows completely deadenylated fragments by RNase H digestion with oligo (dT). The position of the RNA size marker is shown on the left.

How does DAZL cancel the miRNA-mediated repression? In zebrafish embryos, miRNA induces deadenylation of target mRNA [5], [9]. To test whether or not DAZL affects poly(A) tail length, we measured the poly(A) tail length of reporter mRNAs injected into embryos. After injection, GFP reporter mRNA was subjected to RNase H-digestion in the presence of a specific oligonucleotide complementary to the 3′ end of GFP sequence, and the resulting short RNA fragment containing the 3′UTR and poly(A) tail was detected by Northern blotting [5]. We found that the injected GFP-*tdrd7* mRNA lost its poly(A) tail at 3–6 hpf, as expected [5] (Fig. 4A, and data not shown). In contrast, the mRNA possessed a long poly(A) tail in the presence of DAZL (Fig. 4A). The mutant DAZL did not affect poly(A) tail length (Fig. 4A). Therefore, it is likely that DAZL somehow induces polyadenylation, or represses deadenylation, of *tdrd7* mRNA.

We could think of three possible mechanisms by which DAZL might induce polyadenylation of *tdrd7* mRNA. The first possibility is that DAZL activates *tdrd7* translation without affecting poly(A) tail length, and subsequently active translation results in the polyadenylation of *tdrd7* mRNA. The second is that DAZL blocks miR-430 function on *tdrd7* mRNA or its access to *tdrd7* mRNA, and thereby *tdrd7* mRNA is free from the deadenylation mediated by miR-430. The third is that DAZL directly promotes polyadenylation of *tdrd7* mRNA. To evaluate these possibilities, we performed two kinds of experiments.

First, we injected the A-capped reporter mRNA instead of the normal m^7^G-capped mRNA. As the A-capped mRNA cannot be recognized by translation initiation factor (cap binding protein), active translation does not occur while the mRNA is stable in embryos [5]. We found that A-capped GFP-*tdrd7* mRNA was deadenylated, like the m^7^G-capped one, in the absence of DAZL protein; this is consistent with our previous result with GFP-*nanos* mRNA [5] (Fig. 4C). In contrast, DAZL induced the poly(A) tail elongation of A-capped GFP-*tdrd7* mRNA (Fig. 4C). These results exclude the first possibility that active translation induced by DAZL results in the polyadenylation of *tdrd7* mRNA.

Second, we measured the poly(A) tail length of a mutant GFP-*tdrd7* mRNA that lacks the miR-430 target sites, which is not subjected to the repression mediated by miR-430 [5]. As a result, the injected reporter mRNA possessed a long poy(A) tail approximately 150 nt long in the absence of functional DAZL protein (Fig. 4B). In contrast, we found that the poly(A) tail of the reporter mRNA in the presence of DAZL protein was much longer (approximately 200–250 nt long) (Fig. 4B). The results did not support the second possibility that DAZL only blocks deadenylation mediated by miR-430. Rather, it is likely that DAZL promotes the polyadenylation of *tdrd7* mRNA.

### DAZL also controls another germline mRNA, *dazl*, targeted by miR-430

**Figure 5:**
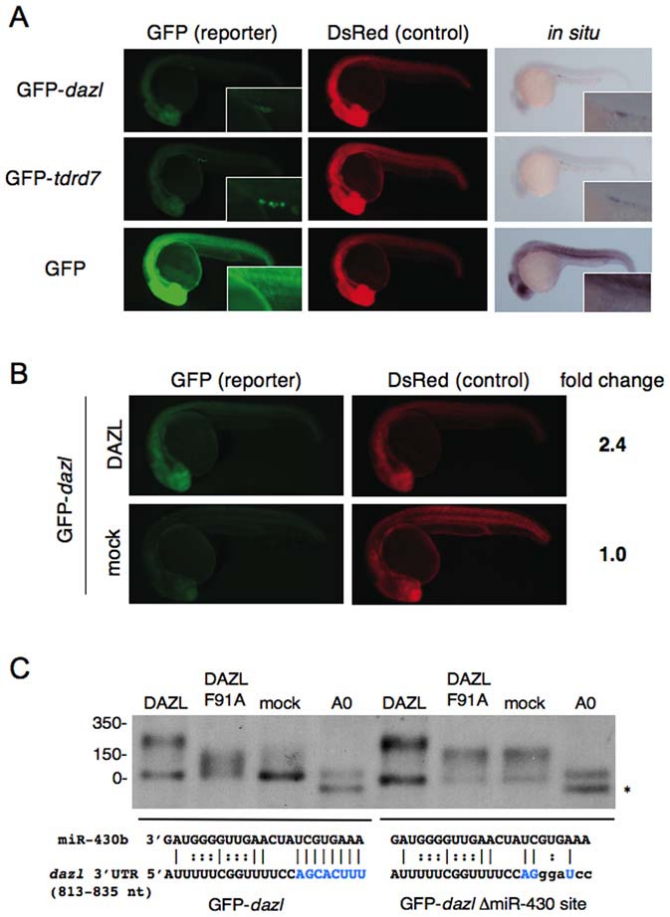
Regulation of *dazl* mRNA by miR-430 and DAZL. (A) GFP-*dazl*, GFP-*tdrd7* or GFP mRNA was injected with DsRed mRNA. GFP and DsRed were analyzed at 24 hpf. Insets show the gonad region. The reporter mRNA was detected by *in situ* hybridization with a GFP probe. (B) GFP-*dazl* and DsRed mRNAs were injected with or without the mRNA encoding Myc-DAZL. GFP and DsRed were analyzed at 24 hpf. Fold change of normalized GFP fluorescence relative to mock control is shown on the right. (C) Poly(A) length of GFP-*dazl* and GFP-*dazl* ΔmiR-430 site mRNAs in the presence or absence of DAZL was analyzed by RNase H-poly(A) assay at 6 hpf. (A0) shows completely deadenylated fragments by RNase H digestion with oligo (dT). The position of the RNA size marker is shown on the left. Sequences of wildtype and mutated miR-430 target sites are shown below. Nucleotides that are complementary with miR-430 seed are shown in blue. Asterisk indicates a non-specific cleavage product of the *dazl* 3′UTR generated with oligo (dT).

Our previous study suggested that DAZL enhanced protein synthesis via the 3′UTR of *dazl* mRNA [15]. So we examined if *dazl* mRNA is also controlled by a combination of DAZL and miR-430. Injection experiments of the GFP mRNA fused with *dazl* 3′UTR (GFP-*dazl*) showed that the 3′UTR restricted DAZL expression to PGCs (Fig. 5A). Overexpression of DAZL enhanced GFP expression from GFP-*dazl* mRNA throughout the embryo (Fig. 5B). Moreover, the reporter mRNA was strongly stabilized in the presence of DAZL (Fig. S3). The *dazl* 3′UTR contains a putative miR-430 target site (corresponding to the nucleoteides +813–835 relative to the stop codon), which has the octamer sequence complementary for the miR-430 seed sequence. As expected, GFP-*dazl* mRNA was subjected to deadenylation (Fig. 5C). A mutant mRNA, GFP-*dazl* ΔmiR-430 site, in which the octamer target site had been base-substituted, had a long poly(A) tail approximately 150 nt long (Fig. 5C). Overexpression of DAZL protein clearly induced the poly(A) elongation (up to 200–250 nt long) of both GFP-*dazl* and GFP-*dazl* ΔmiR-430 site mRNAs (Fig. 5C). Taken together, we concluded that DAZL also counteracts miR-430-mediated repression of *dazl* mRNA, by inducing polyadenylation. This suggests that DAZL promotes its own expression in PGCs, leading to the enhanced expression of the germline-specific proteins, such as TDRD7.

## Discussion

**Figure 6:**
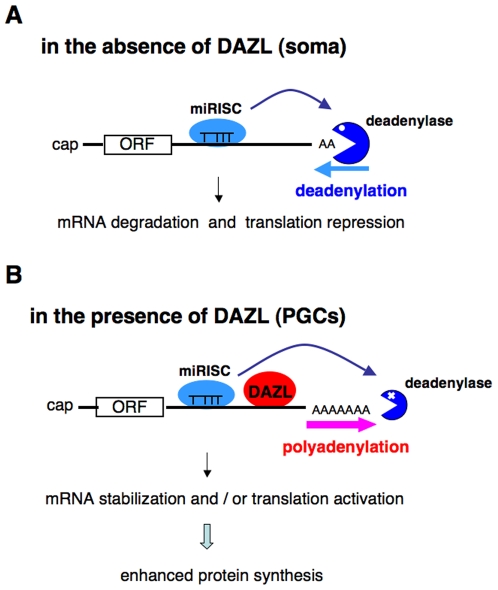
Model for the DAZL action. (A) In the absence of DAZL (e.g. somatic cells), miRISC binds to its target mRNA and induces deadenylation, mRNA degradation and translation repression. (B) In the presence of DAZL (e.g. PGCs), the mRNA bound to DAZL is polyadenylated. This effect can overcome the inhibitory effect by miRISC, leading to the active protein synthesis even in the presence of miRNA.

In this study, we showed that DAZL binds *tdrd7* and *dazl* mRNAs and counteracts the miRNA-mediated repession by inducing polyadenylation in zebrafish (Fig. 6). The DAZ family is evolutionarily conserved and expressed in germline cells. Members of the DAZ family may control germline gene expression by counteracting the repression mediated by miRNAs in various animals.

In *Xenopus* oocytes, it has been shown that DAZL activates the translation of target mRNAs with short poly(A) tails via the recruitment of PABPs [14]. Here we showed that DAZL induces polyadenylation of target mRNA, enhancing mRNA stability and/or translatability (Fig. 6). Thus it raises the possibility that DAZL plays several distinct roles in the target mRNAs. Alternatively, it is possible that DAZL and PABP may cooperatively function to induce polyadenylation on *tdrd7* and *dazl* mRNAs. Future studies will be necessary to clarify the mechanism of how DAZL induces polyadenylation of the germ plasm mRNAs.

In zebrafish and human germline cells, DND suppresses miR-430 function through blocking miRNA accessibility by binding to U-rich mRNA regions (URRs) [10]. The loss of DND function or its target sequences make *nanos* and *tdrd7* 3′UTRs susceptible to miR-430-mediated repression in zebrafish. However, those 3′UTRs appeared to be able to direct PGC-specific protein expression weakly even in the absence of DND function [10]. Moreover, DAZL overexpression strongly induces somatic protein expression from the GFP reporter mRNA fused with the *tdrd7* 3′UTR lacking the DND-binding element, URR (our unpublished observation). Although we cannot exclude the possibility that DAZL can block the association of miRNA with target mRNAs, the induction of polyadenylation by DAZL strongly suggests that DAZL relieves miRNA-mediated repression in a distinct mode from DND. Interestingly, we found that DAZL is not sufficient to promote protein expression through the intact 3′UTR of *nanos1* mRNA (our unpublished observation). Thus, it is likely that DAZL and DND proteins function additively to exclude the miR-430 function on the various germline mRNAs. Loss of function experiments of DAZL will be important to further clarify the interplay of DAZL and DND during germ cell development.

It has been shown that an AU-rich RNA-binding protein HuR relieves CAT-1 mRNA from miR-122 repression upon stress in human liver cells [22]. TRIM-NHL proteins modulate miRNA activity [23], [24]. In addition, some miRNA-mediated repression is relieved by synaptic stimulation of neuron cells [25], [26]. Biogenesis of miRNA is also controlled [27], [28], [29]. A variety of RNA-binding proteins may regulate miRNA function in a spatiotemporal manner or in response to a specific signal during animal development.

## Materials and Methods

### mRNA synthesis and RNA injection

The GFP open reading frame (ORF) and 3′UTR were cloned into the *Bam*HI and *Xba*I sites of pCS2+ [30]. The resulting plasmid was digested with *Xho*I, whereas pCS2+ MT DAZL constructs containing Myc-tag [15] and pCS2+ DsRed [5] were digested with *Asp*718. Using these DNAs as templates, mRNAs were transcribed by the mMessage mMachine SP6 kit (Ambion). To prepare A-capped mRNA, the dinucleotide ApppG (Ambion) was added to the reaction mixture instead of m^7^GpppG [31]. RNA solution contained GFP and DsRed reporter mRNAs at 0.1 mg/ml each, with or without 0.2 mg/ml Myc-DAZL mRNA, was injected into the cytoplasm of one-cell-stage embryos with an IM300 Microinjector (Narishige).

### RNase H-poly(A) assay

The assay was performed essentially as described [5]. Total mRNA was extracted from five embryos and then mixed with 25 pmol oligo DNA complementary to the 3′ end of the GFP ORF sequence (5′-CTCGACCCCGCCTGAC-3′ *).* *The resultant fragment containing the *tdrd7 3′UTR was detected by Northern blotting.

### Image acquisition

Fluorescent images were captured by a Carl Zeiss Ste REO Lumar V12 microscope and an Axio Cam MRm digital camera. To get images of in situ hybridization embryos, we used a Nikon SMZ1500 microscope and a Nikon DXM 1200F digital camera.

### The measurement of fluorescence

The average pixel intensity of a rectangle above the yolk extension (59×118 pixels, total 6962 pixels) was measured using Adobe Photoshop CS4 Extended ver. 11.0. Background signal was measured at dark regions next to the embryo. We measured the intensity of GFP and DsRed fluorescence in three embryos injected. For normalization, GFP signal was divided by DsRed signal. To calculate the fold change of GFP expression with DAZL overexpression, the average of GFP/DsRed ratio was normalized with that of embryos injected without any mRNA encoding DAZL (mock control).

## Supporting Information

Figure S1 DAZL constructs used in this study. (A) Schematic representation of intact and mutant DAZL proteins. (B) Western blotting of Myc-tagged DAZL proteins expressed in the embryos with anti-Myc antibody. Molecular size markers are shown on the left.
(0.09 MB TIF)

Figure S2 DAZL counteracts the miRNA repression. (A) Schematic representation of the 3′UTR of GFP-IPT^miR-204^ *tdrd7* ΔmiR-430 site mRNA. The target site of miR-204 (blue) and the mutated site for miR-430 (gray) are shown. (B) Sequences of wildtype and mutated miR-430 target sites in the *tdrd7* 3′UTR. Nucleotide positions relative to the stop codon were shown above. Nucleotides that basepair with miR-430 seed are indicated in blue. (C) The GFP-IPT^miR-204^ *tdrd7* ΔmiR-430 site and DsRed mRNAs were injected with or without the mRNA encoding Myc-DAZL at the one-cell stage. Subsequently, the miR-204 duplex was injected at the two-cell stage. GFP and DsRed were analyzed at 24 hpf. Fold change of normalized GFP fluorescence relative to mock control is shown on the right.
(0.31 MB TIF)

Figure S3 The effect of DAZL on GFP-*dazl* mRNA stability. (A) In situ hybridization of the injected embryos at 80% epiboly with an antisense probe for GFP. GFP-*dazl* and DsRed mRNAs were injected with or without the mRNA encoding Myc-DAZL. (B) Northern blotting of GFP-*dazl* and DsRed mRNAs injected with or without the mRNA encoding Myc-DAZL.
(0.29 MB TIF)

## References

[1] Knaut H, Pelegri F, Bohmann K, Schwarz H, Nusslein-Volhard C (2000). Zebrafish vasa RNA but not its protein is a component of the germ plasm and segregates asymmetrically before germline specification.. J Cell Biol.

[2] Hashimoto Y, Maegawa S, Nagai T, Yamaha E, Suzuki H (2004). Localized maternal factors are required for zebrafish germ cell formation.. Dev Biol.

[3] Koprunner M, Thisse C, Thisse B, Raz E (2001). A zebrafish nanos-related gene is essential for the development of primordial germ cells.. Genes Dev.

[4] Weidinger G, Stebler J, Slanchev K, Dumstrei K, Wise C (2003). dead end, a novel vertebrate germ plasm component, is required for zebrafish primordial germ cell migration and survival.. Curr Biol.

[5] Mishima Y, Giraldez AJ, Takeda Y, Fujiwara T, Sakamoto H (2006). Differential regulation of germline mRNAs in soma and germ cells by zebrafish miR-430.. Current Biol.

[6] Knaut H, Steinbeisser H, Schwarz H, Nusslein-Volhard C (2002). An evolutionary conserved region in the vasa 3′UTR targets RNA translation to the germ cells in the zebrafish.. Curr Biol.

[7] Wolke U, Weidinger G, Koprunner M, Raz E (2002). Multiple levels of posttranscriptional control lead to germ line-specific gene expression in the zebrafish.. Curr Biol.

[8] Giraldez AJ, Cinalli RM, Glasner ME, Enright AJ, Thomson JM (2005). MicroRNAs regulate brain morphogenesis in zebrafish.. Science.

[9] Giraldez AJ, Mishima Y, Rihel J, Grocock RJ, Van Dongen S (2006). Zebrafish MiR-430 promotes deadenylation and clearance of maternal mRNAs.. Science.

[10] Kedde M, Strasser MJ, Boldajipour B, Oude Vrielink JA, Slanchev K (2007). RNA-binding protein Dnd1 inhibits microRNA access to target mRNA.. Cell.

[11] Xu EY, Moore FL, Pera RA (2001). A gene family required for human germ cell development evolved from an ancient meiotic gene conserved in metazoans.. Proc Natl Acad Sci U S A.

[12] Houston DW, King ML (2000). A critical role for Xdazl, a germ plasm-localized RNA, in the differentiation of primordial germ cells in Xenopus.. Development.

[13] Vasudevan S, Seli E, Steitz JA (2006). Metazoan oocyte and early embryo development program: a progression through translation regulatory cascades.. Genes Dev.

[14] Collier B, Gorgoni B, Loveridge C, Cooke HJ, Gray NK (2005). The DAZL family proteins are PABP-binding proteins that regulate translation in germ cells.. EMBO J.

[15] Maegawa S, Yamashita M, Yasuda K, Inoue K (2002). Zebrafish DAZ-like protein controls translation via the sequence ‘GUUC’.. Genes Cells.

[16] Wilkie GS, Dickson KS, Gray NK (2003). Regulation of mRNA translation by 5′- and 3′-UTR-binding factors.. Trends Biochem Sci.

[17] de Moor CH, Meijer H, Lissenden S (2005). Mechanisms of translational control by the 3′ UTR in development and differentiation.. Semin Cell Dev Biol.

[18] Zeng M, Deng W, Wang X, Qiu W, Liu Y (2008). DAZL binds to the transcripts of several Tssk genes in germ cells.. BMB Rep.

[19] Reynolds N, Collier B, Bingham V, Gray NK, Cooke HJ (2007). Translation of the synaptonemal complex component Sycp3 is enhanced in vivo by the germ cell specific regulator Dazl.. RNA.

[20] Reynolds N, Collier B, Maratou K, Bingham V, Speed RM (2005). Dazl binds in vivo to specific transcripts and can regulate the pre-meiotic translation of Mvh in germ cells.. Hum Mol Genet.

[21] Fox M, Urano J, Reijo Pera RA (2005). Identification and characterization of RNA sequences to which human PUMILIO-2 (PUM2) and deleted in Azoospermia-like (DAZL) bind.. Genomics.

[22] Bhattacharyya SN, Habermacher R, Martine U, Closs EI, Filipowicz W (2006). Relief of microRNA-mediated translational repression in human cells subjected to stress.. Cell.

[23] Hammell CM, Lubin I, Boag PR, Blackwell TK, Ambros V (2009). nhl-2 Modulates microRNA activity in Caenorhabditis elegans.. Cell.

[24] Schwamborn JC, Berezikov E, Knoblich JA (2009). The TRIM-NHL protein TRIM32 activates microRNAs and prevents self-renewal in mouse neural progenitors.. Cell.

[25] Kedde M, Agami R (2008). Interplay between microRNAs and RNA-binding proteins determines developmental processes.. Cell Cycle.

[26] Flynt AS, Lai EC (2008). Biological principles of microRNA-mediated regulation: shared themes amid diversity.. Nat Rev Genet.

[27] Davis BN, Hilyard AC, Lagna G, Hata A (2008). SMAD proteins control DROSHA-mediated microRNA maturation.. Nature.

[28] Piskounova E, Viswanathan SR, Janas M, LaPierre RJ, Daley GQ (2008). Determinants of microRNA processing inhibition by the developmentally regulated RNA-binding protein Lin28.. J Biol Chem.

[29] Viswanathan SR, Daley GQ, Gregory RI (2008). Selective blockade of microRNA processing by Lin28.. Science.

[30] Rupp RA, Snider L, Weintraub H (1994). Xenopus embryos regulate the nuclear localization of XMyoD.. Genes Dev.

[31] Inoue K, Ohno M, Sakamoto H, Shimura Y (1989). Effect of the cap structure on pre-mRNA splicing in Xenopus oocyte nuclei.. Genes Dev.

