# Supplementary figures and images for: DAZL Relieves miRNA-Mediated Repression of Germline mRNAs by Controlling Poly(A) Tail Length in Zebrafish
Source: PLoS One. 2009 Oct 19;4(10):e7513. doi: 10.1371/journal.pone.0007513 (PMC2759513; doi:10.1371/journal.pone.0007513)

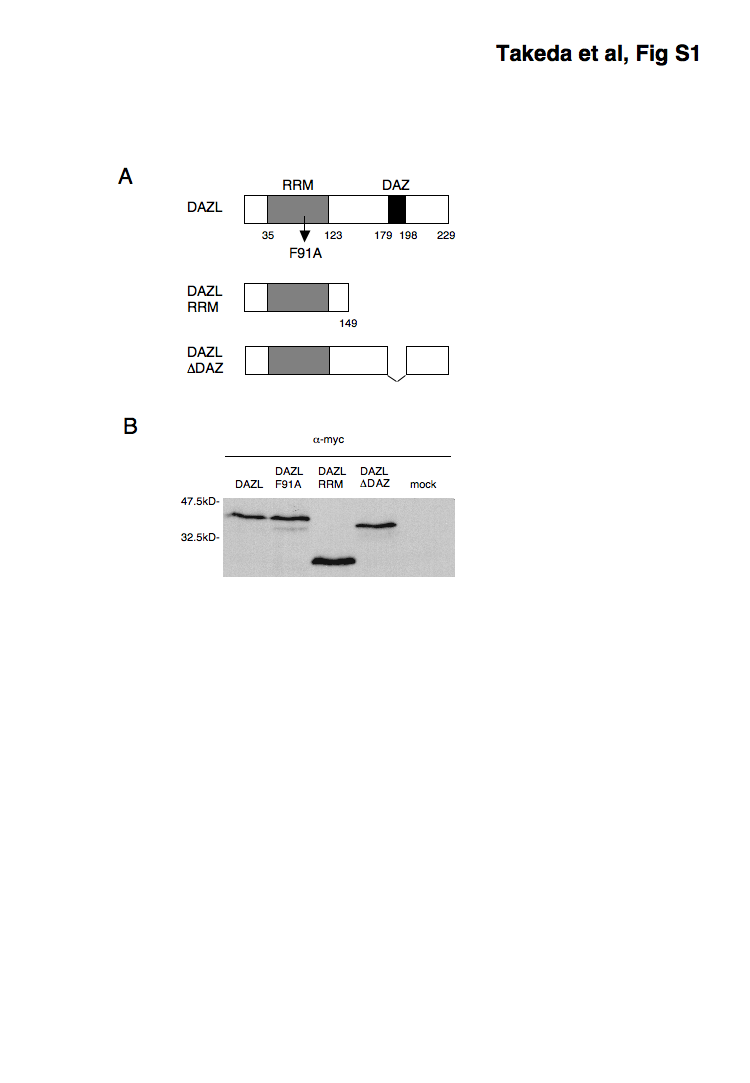

Supplement: Figure S1 — DAZL constructs used in this study. (A) Schematic representation of intact and mutant DAZL proteins. (B) Western blotting of Myc-tagged DAZL proteins expressed in the embryos with anti-Myc antibody. Molecular size markers are shown on the left. (0.09 MB TIF) [file pone.0007513.s001.tif]

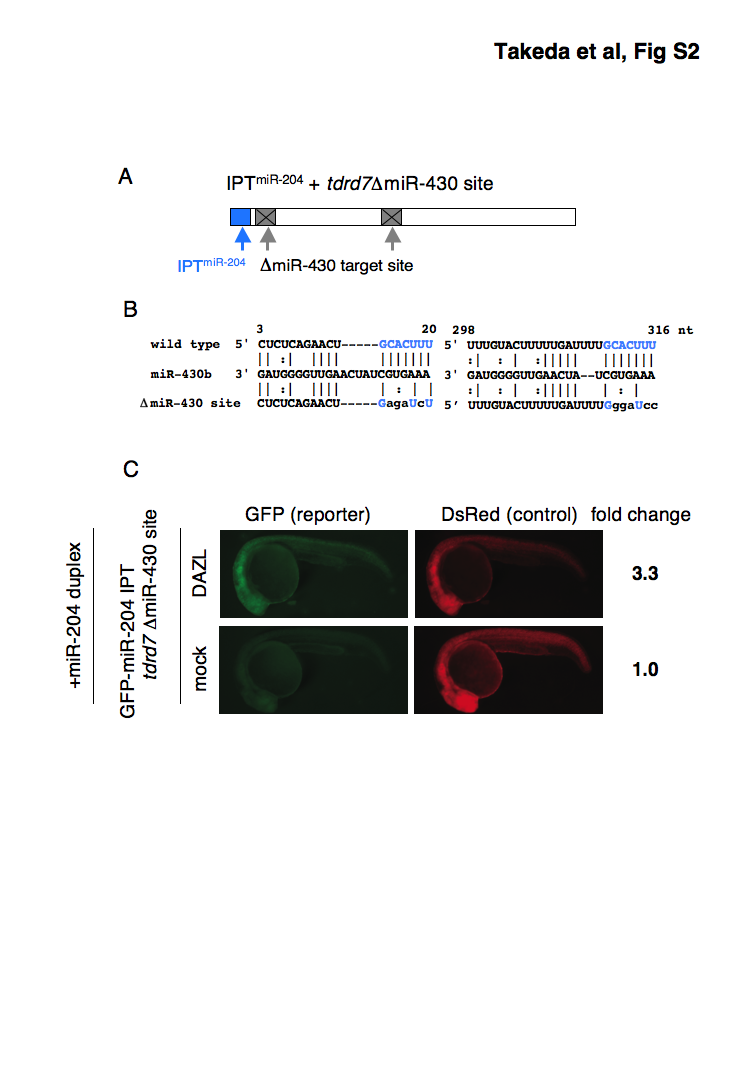

Supplement: Figure S2 — DAZL counteracts the miRNA repression. (A) Schematic representation of the 3′UTR of GFP-IPTmiR-204 tdrd7 ΔmiR-430 site mRNA. The target site of miR-204 (blue) and the mutated site for miR-430 (gray) are shown. (B) Sequences of wildtype and mutated miR-430 target sites in the tdrd7 3′UTR. Nucleotide positions relative to the stop codon were shown above. Nucleotides that basepair with miR-430 seed are indicated in blue. (C) The GFP-IPTmiR-204 tdrd7 ΔmiR-430 site and DsRed mRNAs were injected with or without the mRNA encoding Myc-DAZL at the one-cell stage. Subsequently, the miR-204 duplex was injected at the two-cell stage. GFP and DsRed were analyzed at 24 hpf. Fold change of normalized GFP fluorescence relative to mock control is shown on the right. (0.31 MB TIF) [file pone.0007513.s002.tif]

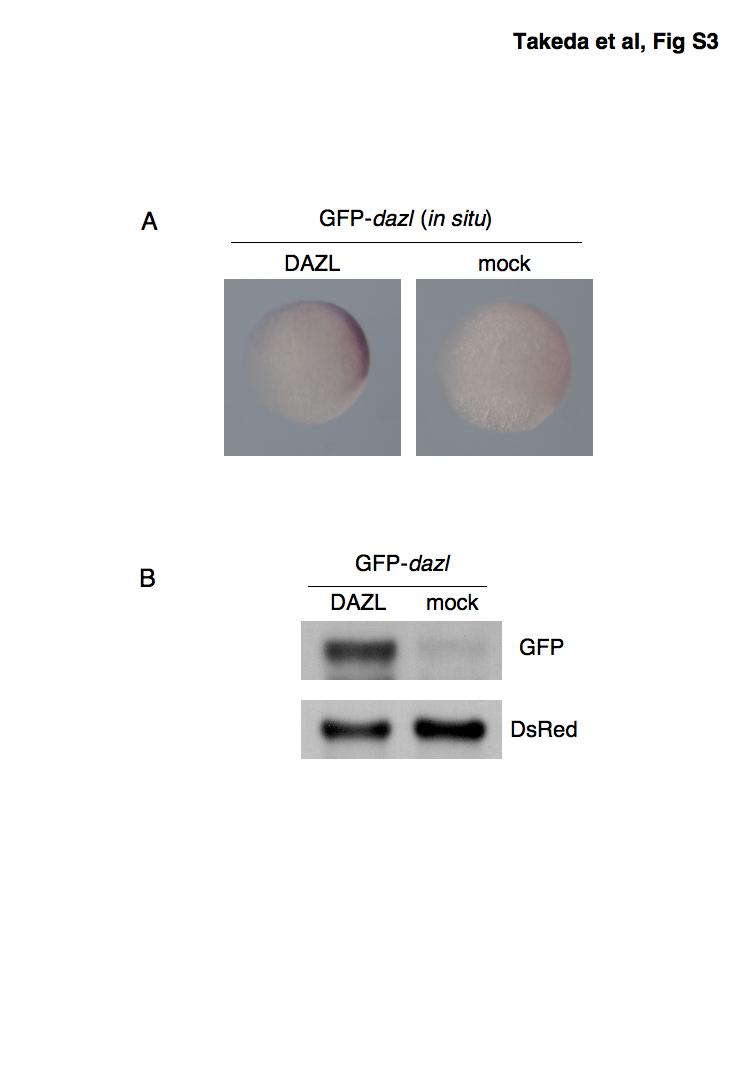

Supplement: Figure S3 — The effect of DAZL on GFP-dazl mRNA stability. (A) In situ hybridization of the injected embryos at 80% epiboly with an antisense probe for GFP. GFP-dazl and DsRed mRNAs were injected with or without the mRNA encoding Myc-DAZL. (B) Northern blotting of GFP-dazl and DsRed mRNAs injected with or without the mRNA encoding Myc-DAZL. (0.29 MB TIF) [file pone.0007513.s003.tif]
